# Chronic THC Inhalation During Early Adulthood Exacerbates Ovariectomy-Induced Bone Loss in a Rat Model of Osteoporosis

**DOI:** 10.3390/biomedicines14081687

**Published:** 2026-07-27

**Authors:** Grace Clouse, Samantha Penman, Aidan Powell, Faisal Sadar, Nihamul Ehan, Isaiah T. Taylor, Ayanna Varma, Michael Hadjiargyrou, David E. Komatsu, Panayotis K. Thanos

**Affiliations:** 1 Behavioral Neuropharmacology and Neuroimaging Laboratory on Addictions (BNNLA), Clinical Research Institute on Addictions, Department of Pharmacology and Toxicology, Jacobs, School of Medicine and Biomedical Sciences, University at Buffalo, 1021 Main Street, Buffalo, NY 14203, USA; graceclo@buffalo.edu (G.C.);; 2 Department of Orthopaedics and Rehabilitation, Renaissance School of Medicine, Stony Brook University, Stony Brook, NY 11794, USA; 3 Biological & Chemical Sciences, New York Institute of Technology, Old Westbury, NY 11568, USA; mhadji@nyit.edu

**Keywords:** delta-9-tetrahydrocannabinol, osteoporosis, cannabis, bone, inhalation, early adulthood

## Abstract

**Introduction:** Osteoporosis is a debilitating bone disease that affects a large segment of the population. Currently, pharmacological treatment options for osteoporosis, including antiresorptive drugs and those that prevent bone loss, are limited. Multiple studies have shown that the endocannabinoid system and cannabis may play a significant role in bone remodeling. As both the use of cannabis and high-quality research on its effects continue to grow, novel medicinal uses of selected components, such as tetrahydrocannabinol (THC), are being investigated. The present study examined the potential effect of chronic THC inhalation during early adulthood on an ovariectomized rodent model of post-menopausal osteoporosis. **Materials and Methods:** Young (26-week-old) adult female rats received daily either THC or air inhalation for 8 weeks. The rats then underwent an ovariectomy (OVX) or sham surgery (Sx) when they were 34 weeks old. They were then euthanized 4 weeks post-OVX, and skeletal tissues were collected for analysis. **Results:** MicroCT analysis of femora showed that THC + OVX rats showed a significant decrease (45%) in trabecular bone volume compared to Air + Sham Sx rats. As expected, the Air + OVX rats also showed a 17% reduction in trabecular bone volume. **Discussion:** Overall, early adulthood THC exposure increased susceptibility to osteoporosis following ovariectomy-induced menopause. This suggests that THC exerts long-lasting effects on bone health, which may exacerbate post-menopausal bone loss.

## 1. Introduction

Among internationally regulated drugs, cannabis, along with its constituents, is the most widely used [1]. Recently, cannabis use has risen to an estimated level of 193 million people worldwide [2]. Medicinal use of the cannabis sativa plant dates back to as early as 2700 B.C.E [3]. The National Surveys on Drug Use and Health (HSDUH) found that of the 12.9% of United States adults reporting cannabis use in the last year, 9.8% of the users use it medicinally [4]. Research on the potential medicinal uses of the cannabis sativa plant, along with its components, has expanded in recent years. Previous studies, both preclinical and clinical, suggest medicinal cannabis use has positive outcomes on relieving generalized pain, neuropathic pain, nausea and vomiting, and appetite, as well as symptoms associated with mood and anxiety disorders [5,6]. Despite its therapeutic effects, cannabis is still classified as a Schedule 1 substance in accordance with the US Drug Enforcement Agency (DEA) [7]. Subsequently, its medicinal uses are not widely accepted, and thus, safety and efficacy for cannabis remain understudied.

Of the over 140 cannabinoids present in cannabis, delta-9-tetrahydrocannabinol (THC) and cannabidiol (CBD) are thought to be the most pharmacologically relevant and are the most studied [8]. The most prominent psychoactive compound, THC, modulates the entire endocannabinoid system through its agonistic effects on the cannabinoid receptors, CB1R and CB2R [9]. While CB1R mainly exerts its effects on the central nervous system, and CB2R primarily on the immune system, several studies showed that both receptors are involved in maintaining bone homeostasis [10,11,12,13,14,15,16]. Preclinical studies examining the role of THC use on bone health reported mixed results for potential therapeutic efficacy in treating fractures, osteoarthritis, and osteoporosis via modulation of osteoblasts and osteoclasts [17,18,19]. The mechanisms by which THC and CBD can affect bone health and outcomes of osteoarthritis were well summarized in a recent review [18]. In a rodent fracture healing model, CBD (5 mg/kg/day I.P.) enhanced maximal load and work-to-failure in male rats [17]. Also, THC (5 mg/kg/day I.P) potentiated the biomechanical improvements induced by CBD (5 mg/kg/day I.P) but was not effective when used alone. In an alveolar bone remodeling model, synthetic THC (Dronabinol 10 mg/kg/day I.P.) was shown to attenuate orthodontic tooth movement by decreasing bone resorption [19]. In humans, short-term (7 days) combined oral THC and CBD has been shown to non-significantly reduce markers of bone resorption in healthy adults [20]. While this may suggest a protective effect of cannabinoids on bone health, the effects of a longer administration period are unclear. The use of cannabinoids as a treatment for bone diseases or conditions can vary widely based on etiology. These mixed findings also highlight the gap in research on the effects of cannabinoids on females and specifically in post-menopausal osteoporosis.

Osteoporosis is a skeletal disorder that weakens bones and increases the risk of fractures [21]. Bone is continuously being remodeled to maintain its integrity and involves the resorption of old bone, mediated by osteoclasts, followed by the formation of new bone, mediated through osteoblasts [22]. If the bone remodeling process is disturbed by favoring resorption, it leads to osteopenia and osteoporosis. As of 2017, an estimated 200 million people have been diagnosed with osteoporosis worldwide. The impact of this disease is overwhelming, with up to one in two women and one in four men over the age of 50 fracturing a bone due to osteoporosis [23]. Moreover, over 6 million osteoporotic hip fractures are projected to occur in the US alone by 2050 [23,24].

While the prevalence of osteoporosis is overwhelming in the elderly population, so is chronic cannabis use during early adulthood. Within this young age group, cannabis is one of the most widely used, and abused, illicit drugs [25,26]. Previous animal and clinical studies have demonstrated that THC use in early adulthood produces lasting changes, including increased impulsivity, attentional issues, memory issues, and anxiety [27,28,29,30,31,32]. Furthermore, other drugs of abuse (psychostimulants) have been shown to alter bone development and bone healing in previous research [33,34,35]. Due to the high prevalence of cannabis use in early adulthood and osteoporosis later in life, combined with previous studies demonstrating the involvement of cannabinoids in bone health [36], our study aimed to investigate the potential role of chronic THC use during early adulthood on subsequent risk of osteoporosis later in life.

## 2. Methods

**Figure 1:**
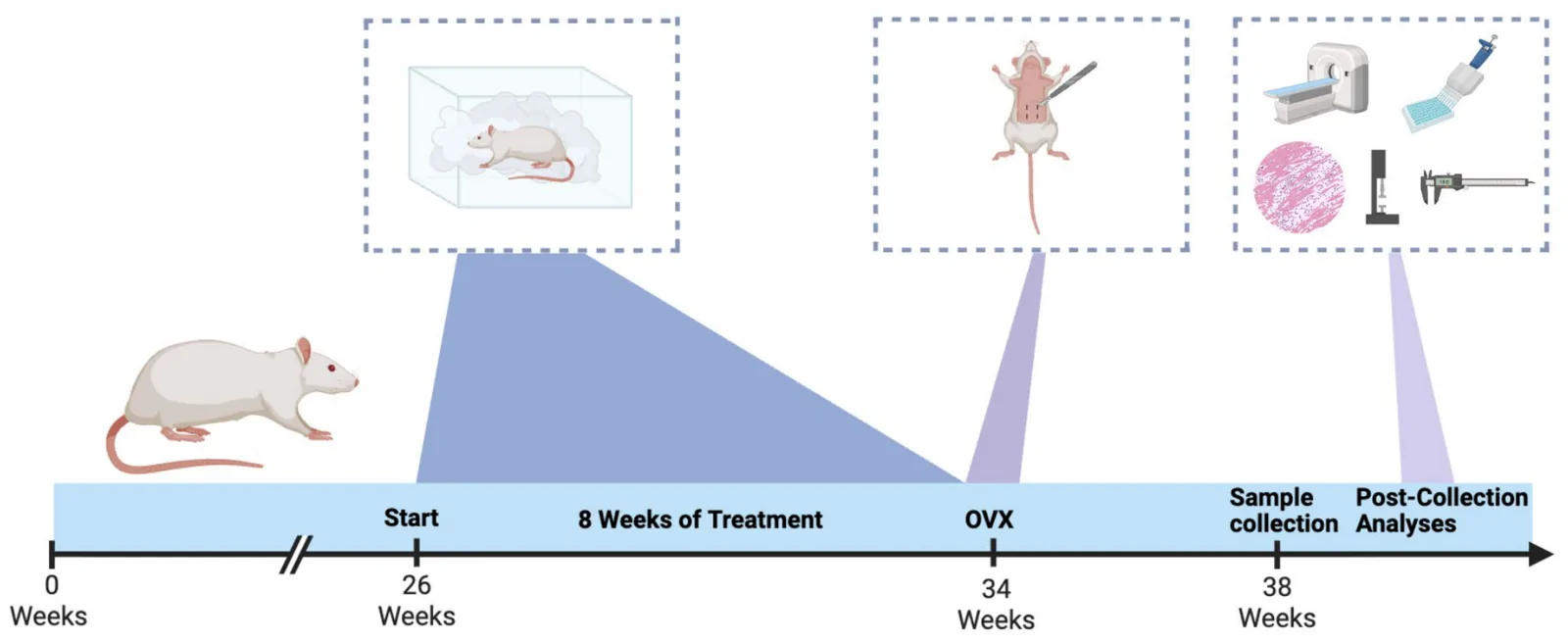
Experimental timeline for procedures. Sprague Dawley rats were exposed to drug treatment (THC or air inhalation) for 8 weeks, and ovariectomy surgeries were performed following drug treatment. The rats were then untreated for 4 weeks and then euthanized, and bones were harvested for analysis.

Animals: Adult female Sprague Dawley (Taconic, Rensselaer, NY) rats were single-housed in a temperature-controlled environment on a 12 h reverse light cycle (lights off 0600-1800). Food and water were provided ad libitum except during drug exposure. All experiments were conducted in compliance with the National Academy of Sciences Guide for the Care and Use of Laboratory Animals and approved by the University at Buffalo Institutional Animal Care and Use Committee. Body weights were recorded weekly for the duration of the study. After acclimation, the rats were randomized by body weight into three treatment groups, and the experiment began at 26 weeks of age. The THC + OVX group (n = 5) was exposed to 8 weeks of THC inhalation, followed by ovariectomy at 34 weeks. The Air + OVX group (n = 5) was exposed to 8 weeks of air treatment, followed by ovariectomy at 34 weeks. The Air + Sham Sx group (n = 5) was exposed to 8 weeks of air treatment followed by sham ovariectomy surgery at 34 weeks. Sample sizes were chosen based on previous publications [37,38]. A summary of the experimental timeline is graphically represented (Figure 1).

### 2.1. Drug

Delta-9-tetrahydrocannabinol (THC) was obtained from the NIDA Drug Supply Program.

THC for inhalation was prepared in a 95% ethanol vehicle from a stock solution of 200 mg/mL.

### 2.2. Treatment

#### Delta-9-tetrahydrocannabinol Inhalation

Rats (n = 5) were placed in a sealed inhalation chamber (2.5 gallon; 16.5 × 11.3 × 5.5 inches) for a 5 min habituation period (Figure 1), as previously described [39,40,41,42,43]. THC solution was vaporized using a Volcano vaporizer (Storz and Bickel, Tuttlingen. Germany). THC solution (0.25 mL) was dispensed onto steel pads for final amounts of 45 mg. After allowing ethanol to fully evaporate, the steel pad was placed in the vaporizer, and the drug was vaporized (at heat setting 9, approximately 226 °C). Vaporized THC was collected in an 8 L plastic balloon. THC vapor was administered into each airtight chamber through a fitted adaptor, which was then sealed, and a 10 min exposure period followed. Exposures were conducted under a fume hood to eliminate any risk of exposure to the investigators. Animals were continuously monitored throughout inhalation. All animals were returned to their home cages once complete. Control animals underwent the same procedure but were exposed to air only.

### 2.3. Ovariectomy

Animals underwent surgery at 34 weeks (Figure 1). Rats (n = 5/group) were bilaterally ovariectomized via dorsal flank incision. A 4 × 4 cm area of the dorsal side of the rat was shaved and sterile-prepped. A 1–2 cm incision was made about 1 cm lateral to the midline, and the skin was blunt-dissected. The fascia and muscle tissue were blunt-dissected to expose the abdominal cavity. Once the ovary and fallopian tube were identified within the adipose tissue, they were then pulled out through this incision. The ovary was isolated and ligated using 4-0 silk monofilament sutures twice, 2 mm apart, on the fallopian tube. The ligations were made as close to the ovary as possible while avoiding any ovarian tissue. The ovary was then removed, the remaining adipose tissue was returned to the abdominal cavity, and the muscle layer was sutured closed with 4-0 absorbable sutures. The skin was then closed with 4-0 absorbable sutures. This procedure was repeated for the second ovary. Following surgery, rats (n = 5) were injected with carprofen (5 mg/kg) for 3 days post-op for pain management and enrofloxacin (5 mg/kg) for 7 days post-op to prevent infection.

### 2.4. Tissue Collection

At the end of the study, the rats were euthanized, and hindlimbs were collected for analyses. Right tibias were isolated, stripped of soft tissues, submerged in 10% neutral buffered formalin (NBF) and stored at 4 °C in a freezer. One day later, the NBF was replaced, and the samples were stored at 4 °C for an additional day. The NBF was then removed, and the samples were transferred to 70% ethanol until further analysis. Left femurs were isolated, stripped of soft tissues, wrapped in saline-soaked gauze and stored at −20 °C. Left tibias were isolated, stripped of soft tissues, submerged in 70% ethanol, and stored at −20 °C.

### 2.5. Caliper Measurements

To assess longitudinal growth, left tibiae were measured with digital calipers (Mitutoyo, Aurora, IL, USA). Length was measured from the top of the tibial condyles to the bottom of the medial malleolus. Diameter was measured along the anterior–posterior (AP) and medial–lateral (ML) axes at the tibial plateau (proximal) and mid-diaphysis (Mid) [33,34,35].

### 2.6. Microcomputed Tomography

Left femora were scanned to evaluate the microstructure and density of trabecular and cortical compartments using a uCT40 (Scanco, Brüttisellen, Switzerland) at a voltage of 55 kV, current of 145 μA, and resolution of 18 μm. Images were then reconstructed using uCT Tomography (Ver. 6.3-4, Scanco). Trabecular analyses were conducted for a metaphyseal region of interest (ROI) beginning 250 slices below the proximal epiphysis and continuing for an additional 115 slices (2.07 mm). Automated scripts were then used to calculate bone fraction (BV/TV), bone mineral density (BMD), trabecular number (Tb. N), trabecular thickness (Tb. Th), trabecular separation (Tb.Sp), and structural model index (SMI). Cortical analyses were conducted for a ROI comprising 100 slices (1.80 mm), centered at the femoral mid-diaphysis, and included cortical volume (Ct.V), periosteal volume (Ps.V), endocortical volume (Ec.V), cortical thickness (Ct.Th), bone mineral density (BMD), and polar moment of inertia (pMOI) [33,34,44,45].

### 2.7. Biomechanical Analyses

Following microCT analyses, the left femora underwent three-point bending to assess biomechanical integrity. Prior to testing, the femora were brought to room temperature and kept hydrated in saline. They were then placed in a custom-designed stainless-steel loading jig with an outer span of 20 mm. A monotonic load to failure was applied along the AP axis at a rate of 20 mm/min, under displacement control, using a MTESTQuattro materials testing system equipped with a 1000 N load cell (Admet, Norwood, MA, USA). Load and displacement were sampled at 100 Hz using the MTESTQuattro software package (Version 3.13.01, Admet). Ultimate force, stiffness, and energy to failure were calculated from force vs. displacement plots using a set of custom-written macros in Excel (Microsoft, Bellevue, WA, USA) [35,46].

### 2.8. Histology

Growth plate height and morphology were assessed in right tibiae. Briefly, the specimens were decalcified in 5% formic acid (Sigma Aldrich, Saint Louis, MO, USA) for ~14 days, dehydrated in ethanol and xylenes, and embedded in paraffin. Coronal sections (5 μm) were then cut with a microtome (Leica, Boston, MA, USA) and stained with Safranin O/ Fast Green. To quantify growth plate height, digital images of the growth plates were acquired from 3 sections/rat at 200× magnification using an inverted microscope (Eclipse E800, Nikon, Melville, NY, USA) equipped with a digital camera (Infinity3 Lumenera, Teledyne, Waterloo, ON, Canada) using Infinity Capture (Ver. 6.5.7, Lumenera). Growth plate height was then measured at three locations (medial, central, and lateral) for 3 sections per rat image using ImageJ (Ver 1.53) [33,44,45,47].

### 2.9. ELISA Analysis

Serum blood levels of estradiol (E2) were measured using ELISA assays (ELISA from MyBioSource, San Diego, CA, USA). The detection range of the E2 ELISA was 1.56–100 pg/mL, and sensitivity was 1.00 pg/mL. In total, seven standards and a blank were used as references. Standards were diluted to 100, 50, 25, 12.5, 6.25, 3.12, and 1.56 pg/mL. To each well, 50 µL of serum sample, standard, or blank was pipetted. Subsequently, 50 µL of biotinylated detection antibody working solution (Concentrated Biotinylated Detection Ab:Biotinylated Detection Ab Diluent = 1:99) was added to each well, followed by a 45 min incubation step at 37 °C. Plates were then washed three times with wash buffer supplied by the manufacturer. Following wash steps, 100 µL of horseradish peroxidase (HRP) conjugate working solution (Concentrated HRP Conjugate:HRP Conjugate Diluent = 1:99) was added to each well. After a 30 min incubation at 37 °C, wells were washed five times with wash buffer; then, 90 µL of substrate reagent was added to each well. After a 15 min incubation at 37 °C, 50 µL of stop solution was added to each well, and plates were read at 450 nm.

### 2.10. Statistical Analysis

Data were averaged by group and are presented in dot plots with lines indicating means. Shapiro–Wilk tests were performed to assess normality, and subsequent comparisons were made using ANOVA or Kruskal–Wallis tests if they passed or failed normality, respectively. Pairwise comparisons for significant differences arising from ANOVA were made using Tukey’s multiple comparisons tests, and those from Kruskal–Wallis were made using Dunn’s multiple comparisons tests. All analyses were performed using Prism (Ver 10.3.1, GraphPad, Boston, MA, USA) at an alpha of 0.05.

## 3. Results

### 3.1. Body Weight Measurements

**Figure 2:**
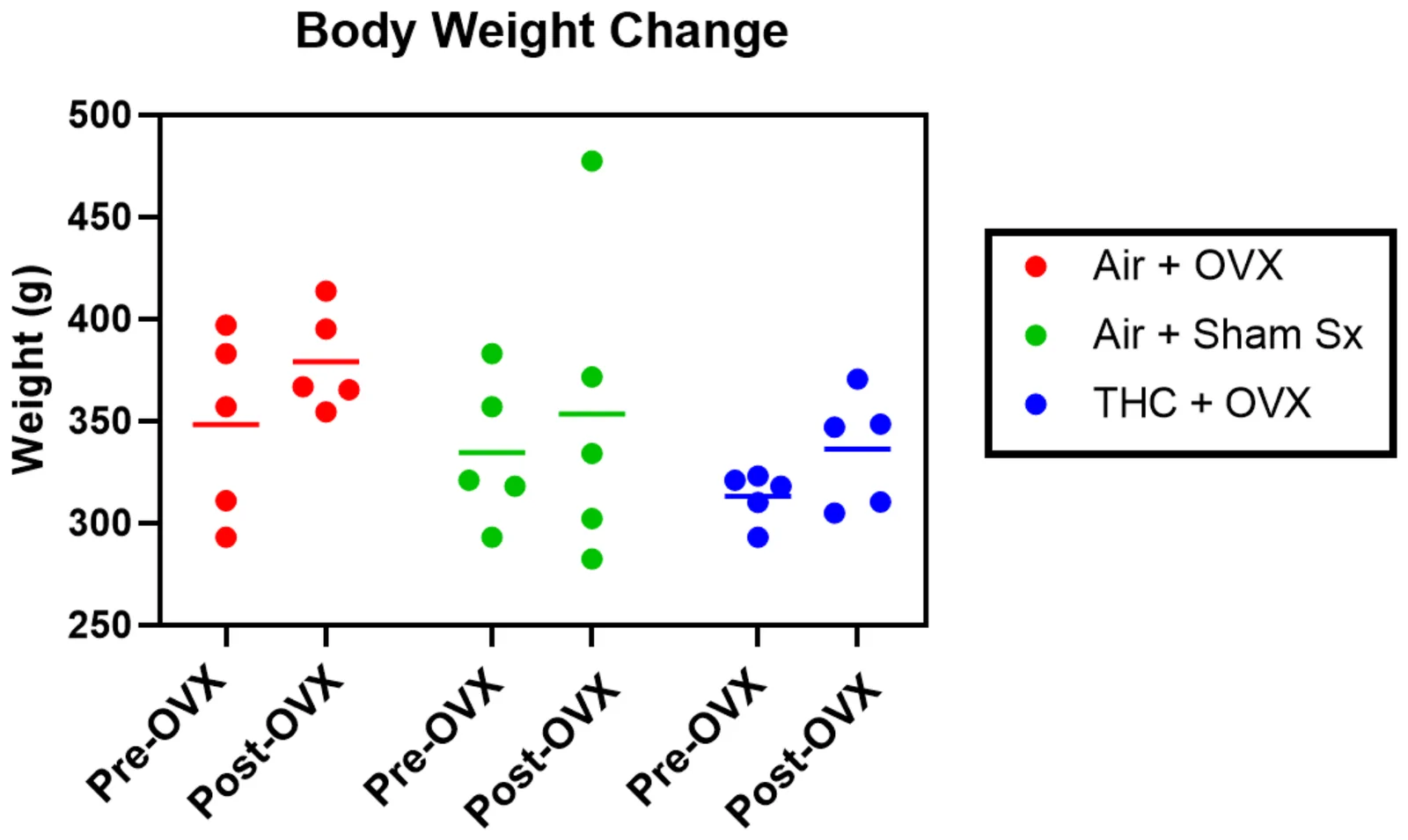
Body weights pre- and post- OVX. Dot plots (mean-line) show body weights of Air + OVX (n = 5) and THC + OVX (n = 5) groups before ovariectomy surgery in grams. No significant difference between groups was found [t(3.504) = 2.489; *p* = 0.0764]. Right box and whisker plots show body weights of Air+ OVX (n = 5), THC + OVX (n = 5), and Air + Sham Sx (n = 5) groups after treatment in grams. No significant difference between groups was found [F(2, 12) = 0.9538; *p* = 0.4127].

No significant differences in body weight were identified comparing Air and THC groups at the time of ovariectomy surgery [t (3.504) = 2.489; *p* = 0.0764] (Figure 2). A one-way ANOVA comparing the body weights of all three groups at the time of euthanasia [Air + OVX, THC + OVX; Air + Sham Sx] found no significant difference between groups [F (2, 12) = 0.9538; *p* = 0.4127; Figure 2].

### 3.2. Caliper Measurements

**Figure 3:**
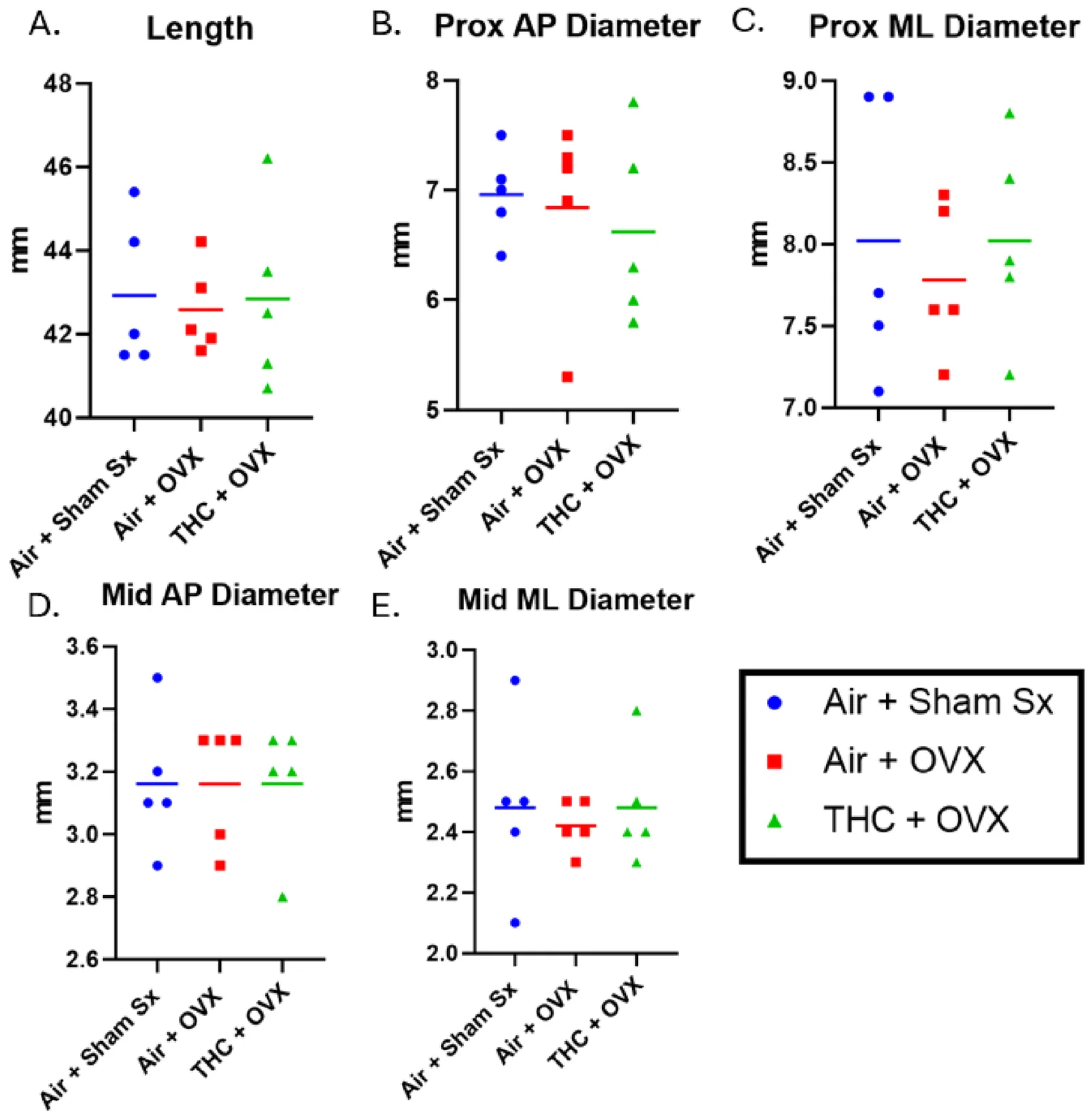
Caliper measurements of tibiae. Dot plots (mean-line) of Air + OVX (n = 5), THC + OVX (n = 5), and Air + Sham Sx (n = 5) at study end. No significant differences in tibia length, proximal AP or MP diameter, or mid-diaphyseal AP or MP diameter were found.

Analysis of tibia length and width via caliper measurements found no significant differences for any measurements (Figure 3).

### 3.3. Microcomputed Tomography and Biomechanical Analysis

**Figure 4:**
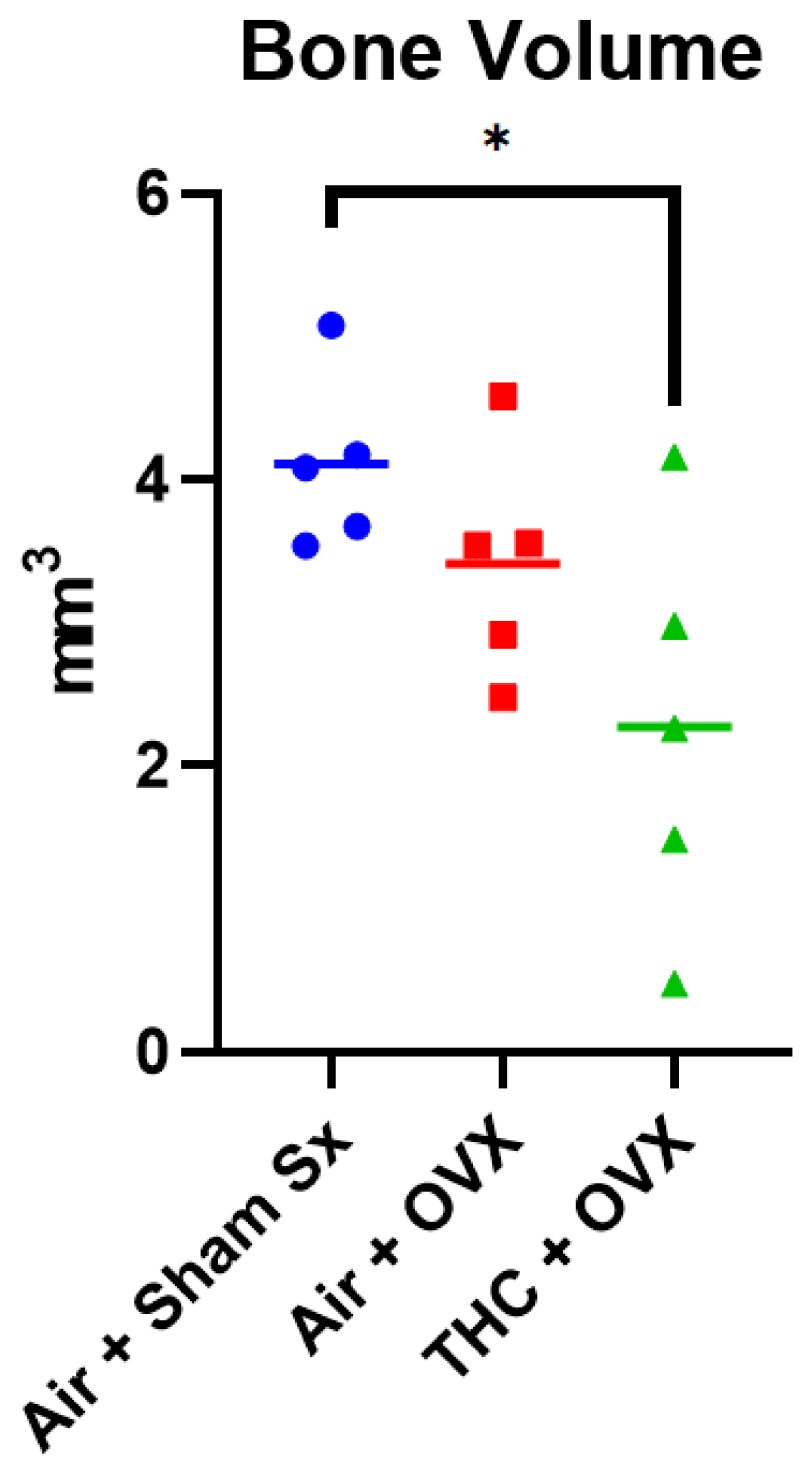
Trabecular microCT measurements. Dot plots (mean-line) showing trabecular microCT parameters from the proximal tibial metaphysis in Air + Sham Sx, OVX + THC, and OVX +Air groups. Significant differences in trabecular volume are displayed. Significant differences between THC treatment rats and controls (ANOVA) are indicated by lines. * *p* < 0.05.

**Figure 5:**
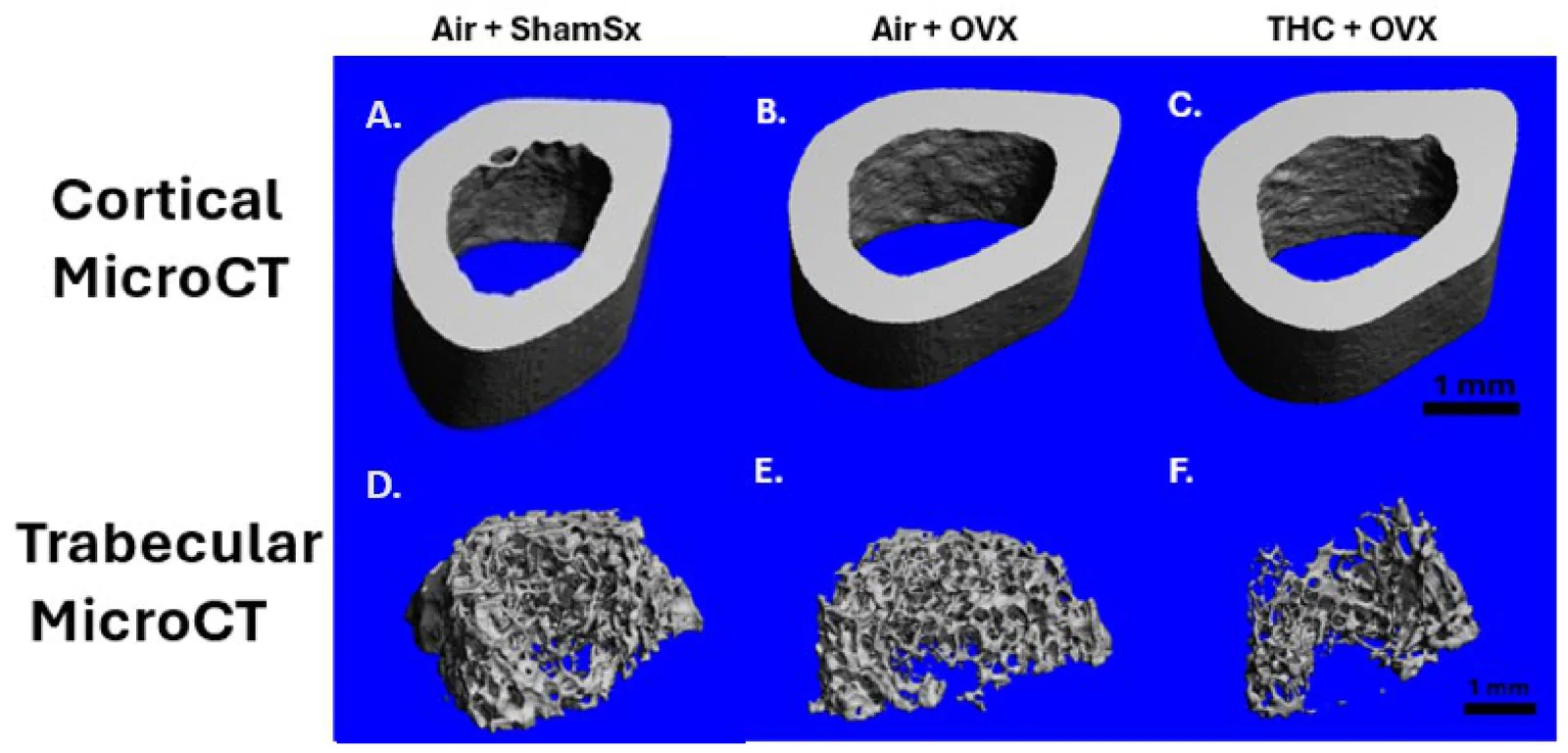
Surface-rendered microCT images. Representative series of femoral microCT images of mid-diaphyseal cortical regions of interest from: (**A**) Air + ShamSx; (**B**) Air + OVX; (**C**) THC + OVX; and proximal metaphyseal trabecular regions of interest from (**D**) Air + ShamSx; (**E**) Air + OVX; (**F**) THC + OVX. Scale bar represents 1 mm.

Analysis of femora by microCT revealed a significant difference in trabecular bone volume (Figure 4), as assessed by ANOVA [F(2, 12) = 4.334; *p* = 0.0383]. Tukey’s multiple comparisons test revealed a 45% bone volume reduction (*p* = 0.0333) as significant in THC + OVX rats compared to Air + Sham Sx rats. While a 17% reduction in trabecular bone volume was also found in Air + OVX rats compared to Air + Sham Sx rats, this did not achieve statistical significance (*p* = 0.5299). No other significant differences were observed between groups for any other microCT parameters (Table 1). Furthermore, no significant differences were found for any biomechanical parameters (Table 1). Surface-rendered microCT images can be seen in Figure 5.

### 3.4. Histology

**Figure 6:**
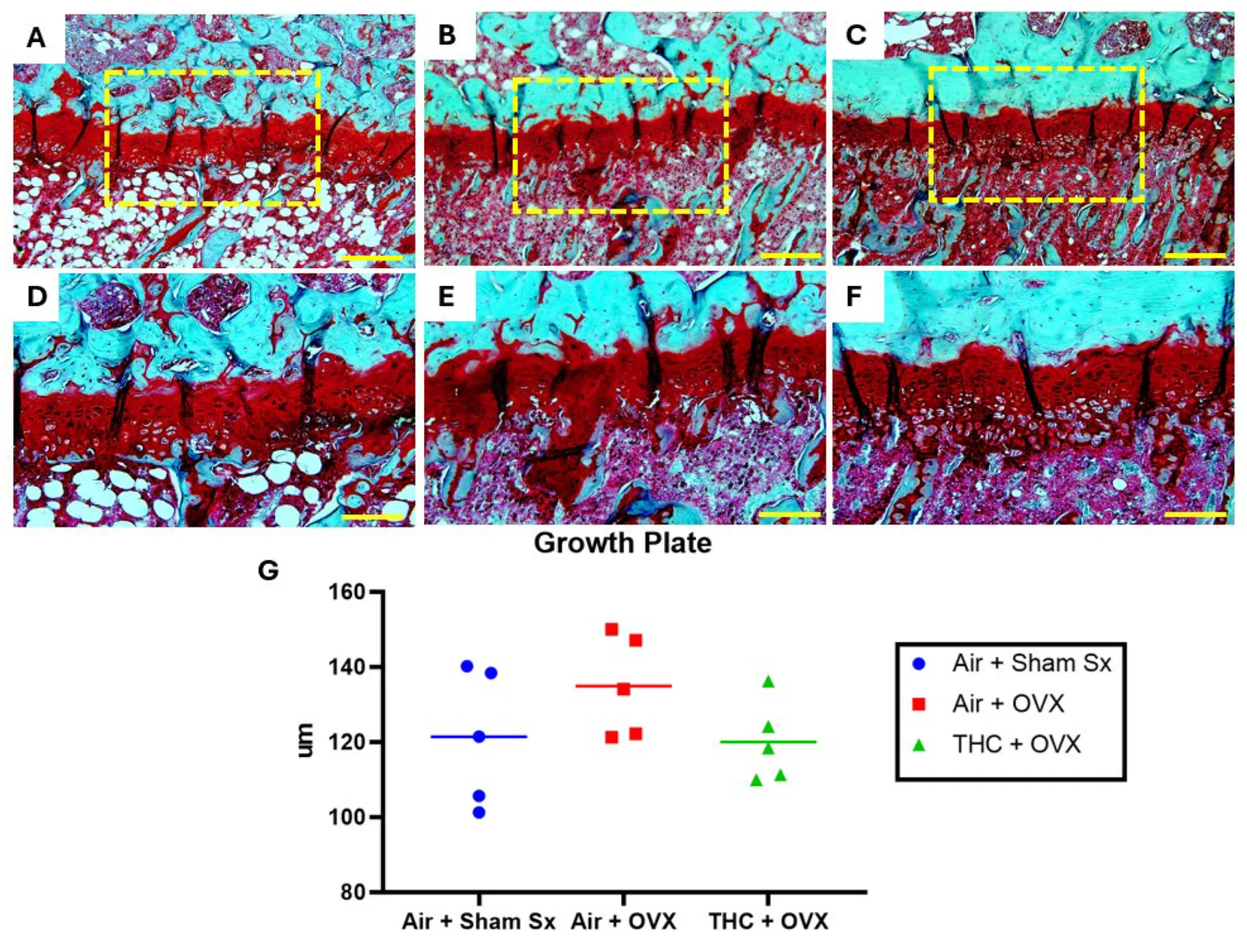
Growth plate histology. Photomicrographs of proximal tibial growth plates stained with Safranin O/Fast Green. (**A**) Air + Sham Sx; (**B**) Air + OVX; and (**C**) THC + OVX at low magnification (100×, scale bars = 200 µm) and (**D**) Air + Sham Sx; (**E**) Air + OVX; and (**F**) THC + OVX at high magnification (200×, scale bars = 100 µm, yellow dashed boxes). (**G**) Dot plots (mean-line) showing growth plate height for each group. Tukey’s multiple comparisons tests revealed these differences were not significant (Air + Sham Sx vs. Air + OVX; *p* = 0.3303) (Air + Sham Sx vs. THC + OVX; *p* = 0.9867).

When we examined the growth plates, we observed normal structural integrity in the Air + Sham Sx and THC + OVX samples (Figure 6A,C,D,F). In contrast, in the presence of Air + OVX, we detected a slightly wider growth plate (Figure 6B,E). Quantitative measures show that compared to Air + Sham Sx, Air + OVX resulted in an 11% increase, while THC + OVX resulted in a 1% decrease in mean growth plate size. However, these differences were not statistically significant (Figure 6G).

### 3.5. E2 ELISA 

**Figure 7:**
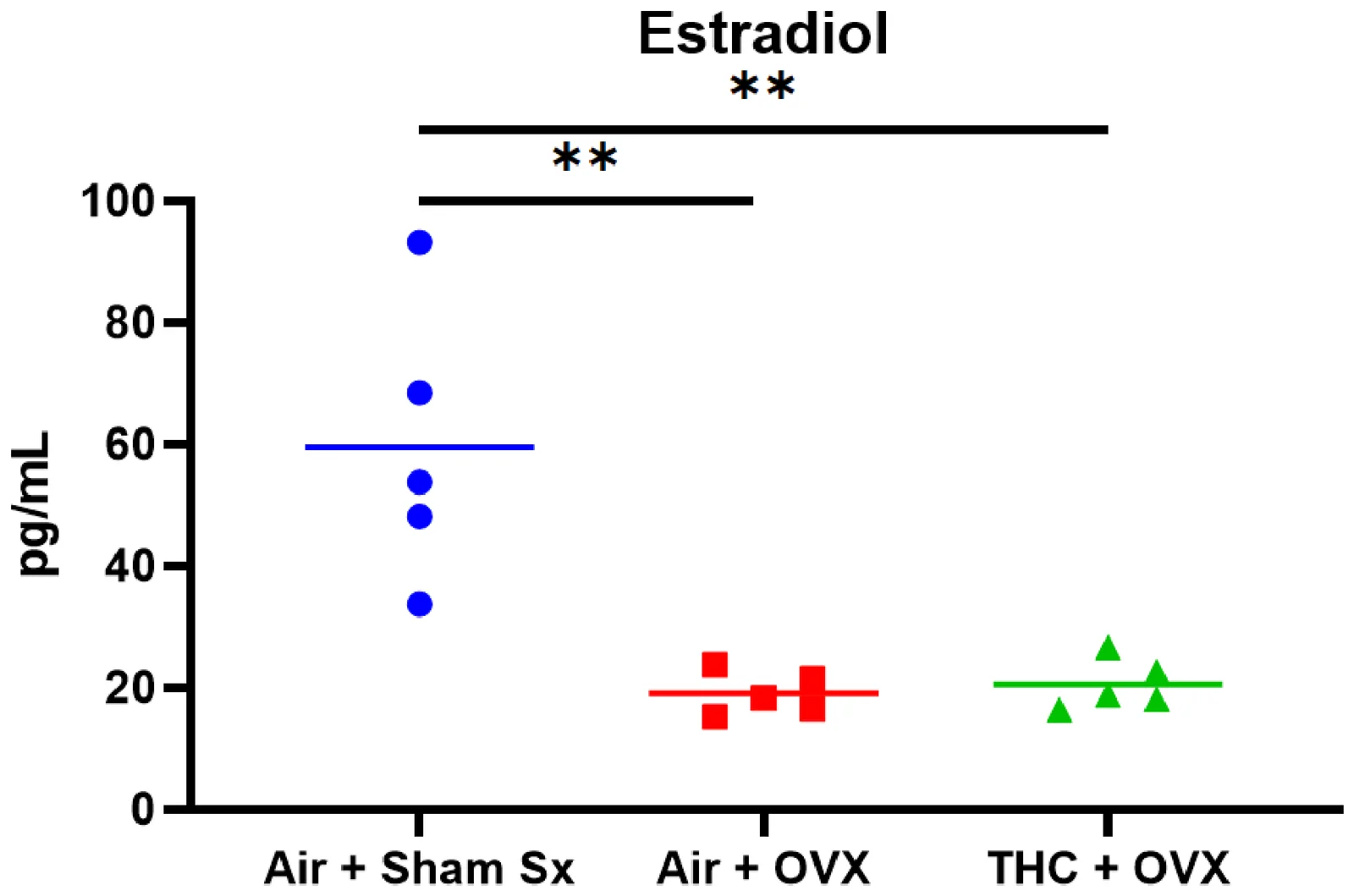
Estradiol (E2) ELISA: Dot plots (mean-line) show mean serum E2 levels in Air + Sham Sx, Air + OVX, and THC + OVX. One-way ANOVA revealed a significant difference between groups [F (2, 12) = 14.58; *p* < 0.0006]. Tukey’s multiple comparisons test revealed significant differences between the THC + OVX and Air + Sham Sx group (*p* < 0.0017) and the Air + OVX and Air + Sham Sx group (*p* < 0.0012). ** *p* < 0.005.

Compared to control animals (Air + Sham Sx), THC + OVX animals showed a 65% significant decrease in mean serum estradiol, while Air + OVX animals showed a 60% decrease (Figure 7). Tukey’s multiple comparisons test revealed significant differences between the THC + OVX and Air + Sham Sx group (*p* < 0.0017) and Air + OVX and Air + Sham Sx group (*p* < 0.0012).

## 4. Discussion

It is difficult to understand how chronic THC use in young adults could impact subsequent risk for osteoporosis, as previous research has resulted in mixed findings [14,48]. This is in part because most heavy cannabis users have other risk factors, such as a higher mean number of alcoholic drinks/day, higher tobacco smoking, and higher illegal drug use [48]. Preclinical research suggests that cannabis smoke inhalation may reduce bone formation and/or increase bone resorption, but smoke exposure could be a confounding factor affecting bone health [49]. Herein, we attempt to narrowly focus on the effects of THC on post-menopausal osteoporosis. Previous research has shown that exposure to psychoactive drugs, including psychostimulants, can negatively impact bone health by reducing bone density, impairing skeletal development, and disrupting bone remodeling processes, in part through increased osteoclast activity and reduced bone integrity [33,34,35,50,51].

Our findings suggest that chronic THC inhalation during early adulthood may promote greater bone loss associated with menopause. Specifically, rats exposed to THC prior to ovariectomy showed a significant reduction in trabecular bone volume compared to control animals that underwent sham surgery, with a significant and approximate 45% decrease in bone volume. While ovariectomy alone produced an expected decrease in trabecular bone volume, this reduction did not reach statistical significance, whereas the decrease observed in the THC + OVX group was significant. Together, these results suggest that prior THC exposure may increase susceptibility to bone loss following estrogen deficiency rather than independently driving bone loss.

Importantly, these effects do not appear to be driven by differences in overall growth or body size. Body weight remained consistent across groups, and no significant differences were observed in tibial length or diameter. Similarly, no significant changes were observed in growth plate morphology, suggesting that THC exposure does not impair general skeletal development but instead may influence bone at the microstructural level, particularly trabecular bone.

Although not statistically significant, the lower body weight observed in the THC-treated group is consistent with prior findings that chronic THC exposure can suppress weight. In adolescent rat models, repeated THC administration has been shown to reduce body weight gain over time, likely due to alterations in metabolic regulation and cannabinoid pharmacokinetics that prolong the physiological effects of THC [52]. These findings suggest that even subtle differences in body weight observed in the present study may reflect underlying metabolic effects of chronic THC exposure rather than random variation.

Previous studies examining the relationship between cannabis use and bone health have yielded mixed results. Some clinical studies have linked heavy cannabis use to lower bone density and increased fracture risk [14], while others suggest that certain cannabinoids may have therapeutic potential. For example, CBD has been shown to enhance fracture healing in rodent models, while THC alone was not effective, but it potentiated these effects when administered in combination [17]. These studies, however, vary widely in terms of experimental design, route of administration, age, and dosage, which likely contribute to the inconsistency in outcomes.

The purpose of the ovariectomy model used in this study was to mimic post-menopausal osteoporosis by removing the protective effects of estrogen on bone homeostasis. In the previous literature, estrogen deficiency has been shown to increase osteoclast activity while reducing osteoblast function, shifting the balance toward bone resorption [21]. In this context, our results suggest that prior THC exposure may increase the vulnerability of the skeletal system to these changes, resulting in more pronounced bone loss following OVX-induced estrogen depletion. This was confirmed by the ELISA analysis, which demonstrated a significant reduction (~60–65%) in serum estradiol levels in both OVX groups compared to sham controls. Notably, no significant differences were observed between the THC + OVX and Air + OVX groups, indicating that THC exposure did not independently alter circulating estradiol levels. Together, these findings confirm that the OVX procedure effectively induced estrogen deficiency and validated the use of this model to study menopause-associated bone loss.

Previous research suggests that the endocannabinoid system (ECS) plays a central role in mediating the effects of post-menopausal osteoporosis. Estrogen deficiency has been shown to alter endocannabinoid signaling, including changes in CB1 and CB2 receptor expression, suggesting that loss of estrogen may dysregulate this system and contribute to increased bone resorption [11,13]. In OVX rat models, estradiol administration has been shown to upregulate CB1 and CB2 receptor expression, as well as levels of the endocannabinoid anandamide (AEA), indicating that estrogen positively regulates ECS activity [53]. In addition, estradiol has been shown to inhibit osteoclast activity through upregulation of CB2 receptor expression, suggesting a protective role of estrogen-mediated ECS signaling in limiting bone resorption [54]. Therefore, we hypothesize that loss of estrogen may contribute to dysregulation of the ECS, promoting increased bone resorption and reduced skeletal integrity.

The ECS likely plays a central role in regulating bone remodeling through its effects on osteoblast and osteoclast activity. Both CB1 and CB2 receptors are expressed in bone tissue and contribute to maintaining the balance between bone formation and resorption [11,13]. CB1 signaling is particularly important for osteoblast differentiation and bone formation. Pharmacological inhibition of CB1 using AM251, a selective CB1 receptor antagonist, has been shown to reduce osteoblast differentiation and bone nodule formation while increasing adipocyte accumulation within bone marrow, a phenotype associated with post-menopausal osteoporosis [55]. Additionally, CB1 expression in bone has been shown to increase with age, suggesting a role in age-related skeletal remodeling [55].

Disruption of CB1 signaling also affects key regulators of bone turnover. In aged and osteoporotic rat models, pharmacological CB1 blockage has been shown to decrease serum osteoprotegerin (OPG), a decoy receptor that inhibits osteoclast differentiation, and increase receptor activator of nuclear κB ligand (RANKL), a key activator of osteoclast formation activity, thereby promoting osteoclastogenesis and bone resorption. These changes are accompanied by reductions in BMD, BMC, cortical thickness, and trabecular bone density [56]. These findings indicate that CB1 signaling is essential for maintaining bone mass and structural integrity, backing our hypothesis of the underlying mechanism by which THC affects bone health.

Chronic exposure to THC has also been shown to impair CB1 receptor function through receptor desensitization and altered downstream signaling. Specifically, chronic THC exposure eliminates CB1-dependent synaptic responses and reduces receptor-mediated signaling, suggesting functional desensitization or uncoupling of CB1 receptors [57]. Consistent with these findings, chronic THC treatment has also been shown to significantly reduce cannabinoid receptor-stimulated G-protein activation across multiple brain regions, indicating widespread desensitization of cannabinoid signaling pathways [58]. These findings support the conclusion that prolonged THC exposure leads to impaired ECS signaling rather than sustained receptor activation.

In the context of bone, such desensitization would be expected to disrupt normal regulation of osteoblast and osteoclast activity, favoring increased bone resorption and reduced bone formation. When combined with estrogen deficiency, which independently alters ECS signaling and promotes osteoclast activity, these effects may act synergistically to impair bone remodeling. Trabecular bone is particularly sensitive to changes in remodeling dynamics due to its high metabolic activity, increased surface area, and rapid annual turnover compared to cortical bone [59]. We hypothesize that this interaction contributes to the enhanced and selective trabecular bone loss observed in the THC + OVX group of the current study.

Interestingly, despite the reduction in trabecular bone volume in the THC + OVX group, no significant differences were found in the cortical bone parameters or biomechanical strength. This pattern suggests that the effects of THC may be more specific to trabecular bone, which is more metabolically active and responsive to hormonal and physiological changes than cortical bone [59]. This is consistent with established patterns of bone loss in osteoporosis, where remodeling occurs primarily on bone surfaces of trabecular bone due to its greater surface area, resulting in trabecular bone undergoing the earliest and most pronounced loss [60]. Since trabecular bone loss is a key early feature of osteoporosis [12], these findings further support the relevance of our results to disease progression.

Additionally, the absence of significant changes in growth plate structure further indicates that THC exposure does not disrupt bone formation during development but rather affects processes related to bone remodeling and maintenance. This finding is consistent with established patterns of skeletal maturation in rodents, where longitudinal bone growth largely ceases by approximately 26 weeks of age despite the continued presence of a growth plate [61]. This functional cessation of growth is attributed to structural and cellular changes within the growth plate, indicating loss of hypertonic chondrocytes, reduced cellular proliferation, and increased irregularity and acellularity, which collectively impair coordinated longitudinal expansion [61]. Therefore, because THC exposure in this study occurred after the period of active longitudinal growth, it is unlikely to have impacted growth-plate-driven bone elongation, further supporting the conclusion that the observed effects are specific to bone remodeling processes rather than skeletal development.

Translationally, these findings suggest that THC exposure after skeletal maturity, such as in late adolescence or adulthood, may be more likely to affect bone quality through remodeling processes rather than impairing linear growth. In the context of post-menopausal women, this may be particularly relevant, as estrogen deficiency already predisposes to increased bone resorption [21], potentially exacerbating the impact of THC on skeletal integrity.

Limitations and Future Directions: There are a few limitations to the current study, including a lack of direct mechanistic limitations, such as molecular analyses of cannabinoid receptors, bone remodeling markers and osteoblast/osteoclast activity. While we have provided a hypothetical mechanism supported by the previous literature, future studies should directly interrogate the mechanisms behind the observed change in trabecular bone. Another limitation is that this experiment lacked a treatment group receiving sham surgery and chronic THC. Therefore, the skeletal effect of THC alone cannot be definitively stated. Lastly, although measurements of body weight were recorded throughout the experiment, we did not measure feeding patterns or other metabolic metrics, which may have contributed to altered body composition and/or bone health. As previously outlined, our data shows deficits in trabecular bone as the most sensitive bone compartment. Future studies should test if observed deficits persist and/or worsen over longer experimental periods. In our model, rodents were only in a “menopausal” state for 8 weeks. Longer menopausal duration may result in different effects.

Conclusion: In our model of post-menopausal osteoporosis utilizing ovariectomy to induce an estrogen-deficient state, administration of THC vapor during young adulthood (prior to estrogen deficiency) increased susceptibility to trabecular bone volume loss. Changes in the trabecular compartment are concerning, as early osteoporotic changes are most pronounced in this bone type. Our results suggest a detrimental effect of THC on bone health; however, future research should both replicate our findings and investigate the underlying molecular mechanism(s) responsible for the observed changes.

## Figures and Tables

**Table 1:** Tibia microCT and biomechanical analysis between groups. Groups were compared by ANOVA; multiple comparisons were made between groups using Tukey’s HSD. Resulting *p*-values are shown. ^a^ indicates data was found to be parametric, assessed by Shapiro–Wilk tests. ^b^ indicates data was non-parametric. Significant pairwise comparisons were also reported. A significant difference in bone volume was found [F(2, 12) = 4.334; *p* = 0.0383]. Pairwise comparison found a significant decrease in bone volume between THC treatment rats and controls. No other significant differences were observed.

		Air + ShamSx		Air + OVX		THC + OVX			Pairwise Comparisons
		Average	StDev	Average	StDev	Average	StDev	*p*-Value	
**Trabecular**	Total Volume	18.08	2.19	17.55	2.15	14.38	3.25	*p* = 0.0878 ^a^	-
	Bone Volume (mm^3^)	4.11	0.60	3.41	0.79	2.27	1.40	*p* = 0.0383 ^a^	Air + Sham Sx vs. THC + OVX
	Bone Volume Fraction	0.23	0.03	0.20	0.05	0.15	0.07	*p* = 0.1297 ^a^	-
	Connectivity Density (1/mm^3^)	107.13	68.07	62.68	20.08	44.51	36.90	*p* = 0.2826 ^b^	-
	Structural Model Index	1.29	0.41	1.40	0.41	1.81	0.54	*p* = 0.2092 ^a^	-
	Trabecular Number (1/mm)	3.24	0.46	2.71	0.87	2.20	1.00	*p* = 0.1677 ^a^	-
	Trabecular Thickness (mm)	0.09	0.01	0.09	0.01	0.09	0.00	*p* = 0.1949 ^a^	-
	Trabecular Spacing (mm)	0.36	0.05	0.46	0.14	0.60	0.31	*p* = 0.1804 ^a^	-
	Tissue Mineral Density	354.31	33.65	320.34	60.42	282.51	60.50	*p* = 0.5140 ^a^	-
	BMD (mg HA/cm^3^)	931.61	16.92	924.83	12.11	944.53	23.94	*p* = 0.5629 ^a^	-
**Cortical**	Bone Volume (mm^3^)	15.51	1.89	14.66	0.75	14.79	0.89	*p* = 0.5442 ^a^	-
	Cortical Thickness (mm)	0.74	0.05	0.69	0.05	0.71	0.03	*p* = 0.3127 ^a^	-
	BMD (mg HA/cm^3^)	1189.81	11.65	1182.64	9.15	1191.71	9.58	*p* = 0.3634 ^a^	-
	Endosteal Volume (mm^3^)	8.75	1.00	9.57	1.39	8.91	1.13	*p* = 0.5255 ^a^	-
	Periosteal Volume (mm^3^)	24.21	2.88	24.19	1.02	23.66	1.70	*p* = 0.8896 ^a^	-
	Polar Moment of Inertia (mm^4^)	17.00	3.92	16.29	1.00	15.92	2.12	*p* = 0.8091 ^a^	-
	Imax [mm^4^]	10.49	2.44	9.95	0.49	9.78	1.57	*p* = 0.7904 ^a^	-
	Imin [mm^4^]	6.51	1.52	6.34	0.62	6.14	0.56	*p* = 0.8485 ^a^	-
**Biomechanics**	Energy to Failure (mJ)	59.31	7.74	57.28	6.20	67.15	21.65	*p* = 0.5072 ^a^	-
	Stiffness (N/mm)	209.37	13.23	201.62	8.48	194.82	17.68	*p* = 0.2796 ^a^	-
	Ultimate Force (N)	141.68	8.33	138.65	9.33	141.78	17.81	*p* = 0.9057 ^a^	-
	Failure Force (N)	141.60	8.43	138.64	9.33	141.45	17.68	*p* = 0.9161 ^a^	-
	Yield Force (N)	124.14	4.56	127.74	12.79	128.76	10.22	*p* = 0.7424 ^a^	-

## Data Availability

The data are available from the corresponding author upon reasonable request.
